# A Naturally Occurring Mutation in *ropB* Suppresses SpeB Expression and Reduces M1T1 Group A Streptococcal Systemic Virulence

**DOI:** 10.1371/journal.pone.0004102

**Published:** 2008-12-31

**Authors:** Andrew Hollands, Ramy K. Aziz, Rita Kansal, Malak Kotb, Victor Nizet, Mark J. Walker

**Affiliations:** 1 School of Biological Sciences, University of Wollongong, Wollongong, New South Wales, Australia; 2 Department of Microbiology and Immunology, Faculty of Pharmacy, Cairo University, Cairo, Egypt; 3 The VA Hospital, Memphis, Tennessee, United States of America; 4 The Department of Molecular Genetics, Biochemistry and Microbiology, The University of Cincinnati, College of Medicine, Cincinnati, Ohio, United States of America; 5 Department of Pediatrics, University of California San Diego, La Jolla, California, United States of America; 6 Skaggs School of Pharmacy and Pharmaceutical Sciences, University of California San Diego, La Jolla, California, United States of America; Columbia University, United States of America

## Abstract

Epidemiological studies of group A streptococcus (GAS) have noted an inverse relationship between SpeB expression and invasive disease. However, the role of SpeB in the course of infection is still unclear. In this study we utilize a SpeB-negative M1T1 clinical isolate, 5628, with a naturally occurring mutation in the gene encoding the regulator RopB, to elucidate the role of RopB and SpeB in systemic virulence. Allelic exchange mutagenesis was used to replace the mutated *ropB* allele in 5628 with the intact allele from the well characterized isolate 5448. The inverse allelic exchange was also performed to replace the intact *ropB* in 5448 with the mutated allele from 5628. An intact *ropB* was found to be essential for SpeB expression. While the *ropB* mutation was shown to have no effect on hemolysis of RBC's, extracellular DNase activity or survival in the presence of neutrophils, strains with the mutated *ropB* allele were less virulent in murine systemic models of infection. An isogenic SpeB knockout strain containing an intact RopB showed similarly reduced virulence. Microarray analysis found genes of the SpeB operon to be the primary target of RopB regulation. These data show that an intact RopB and efficient SpeB production are necessary for systemic infection with GAS.

## Introduction

*Streptococcus pyogenes* (group A streptococcus; GAS) is a Gram-positive, human-specific pathogen responsible for over 500,000 deaths each year [1]. Severe invasive GAS infections such as necrotizing fasciitis account for approximately 30% of these deaths, and the incidence of such acute conditions has been on the rise since the mid 1980's [2]. This resurgence has been paralleled by the emergence of a globally disseminated GAS cone belonging to serotype M1T1 [3]–[5]. While the M1T1 GAS has become the most common cause of streptococcal pharyngitis, this clone is also overrepresented in cases of severe invasive disease [6], [7].

Studies of M1T1 clinical isolates from invasive disease cases have revealed an inverse relationship between expression of the extracellular cysteine protease SpeB and clinical severity [8]. The existence of a SpeB-negative invasive phenotype has been hypothesized that results from mutations in the regulator covR/S [9]. SpeB is a secreted cysteine protease initially expressed as 40 kDa zymogen which is then converted to the 28 kDa active form by autocatalytic processing [10]. SpeB is known to cleave numerous host proteins including components of the extracellular matrix, cytokine precursors, immunoglobulins and antimicrobial peptides [11]–[13], which could interfere with host immune functions. However, SpeB has also been shown to cleave a range of GAS proteins such as the fibrinogen-binding M1 protein [14], [15], various superantigens [16], [17], the secreted plasminogen activator streptokinase [18] as well as the DNase Sda1 [17], and thus possibly interfere with the proven virulence functions of these bacterial factors. The precise role(s) of SpeB throughout the course of infection are undoubtedly complex, and not surprisingly, different studies using different *in vivo* animal models have produced seemingly contradictory results [19]–[21].

In this study we examined the effect of a natural mutation in the gene encoding the regulator RopB (also known as Rgg [22]) identified in a SpeB-negative GAS clinical isolate. RopB is a GAS transcriptional regulator that has been shown to be essential for expression of SpeB and binds directly to the promoter region of *speB* [23], [24]. In studies performed in different GAS serotype strains, RopB has variably been suggested to be involved in the regulation of other GAS genes including those associated with metabolism of non-glucose carbohydrates and amino acids [25], [26], response to thermal and oxidative stress [25], [27] and the expression of virulence factors including DNases (MF-1 and MF-3) and hemolysins (streptolysin S and streptolysin O) [26], [28], [29]. Subsequent investigations into the effect of RopB on virulence have yielded differing results. A study utilizing a zebrafish intramuscular infection model with serotype M5 GAS showed that inactivation of RopB resulted in decreased virulence [30], whereas a study utilizing a murine intraperitoneal infection model with serotype M49 GAS showed that inactivation of RopB resulted in increased virulence [27].

While such global differences in virulence effects could in part result from the differing animal models used, it may also reflect strain-specific variation in the RopB regulon. For example, separate studies have shown *ropB* mutation to have either no effect on hemolysis and DNase activity or, alternatively, to increase expression of hemolysin and DNase-encoding genes and the associated phenotypic activities [23], [26]. This strain-specific variation is highlighted in a recent work by Dmitriev *et al.* [29] that shows inter- and intra-serotypic variation in the transcriptome of *ropB* mutant GAS, with only members of the SpeB operon being commonly regulated in all strains tested.

It is in the light of the current uncertainty surrounding RopB and its role in virulence that we sought to investigate the role of this transcriptional regulator in the serotype M1T1 GAS background that is the leading agent of severe human infection. This analysis begins with a naturally-occuring mutation in *ropB* identified in one such strain.

## Materials and Methods

### Bacterial strains, media and growth conditions

The M1T1 GAS clinical isolates, 5448 and 5628, used in this study have been previously described [31]. Both 5448 and 5628 are *speA*-positive M1T1 strains that were isolated from patients with STSS that were recruited through an ongoing population-based surveillance for invasive GAS infections in Ontario, Canada. Both strains were determined to be derived from the same clone as detailed elsewhere [3]. GAS strains were grown in Todd-Hewitt broth containing 1% yeast extract (THY), or on Todd-Hewitt agar plates (THA). *Escherichia coli* were grown in Luria-Bertani broth (LB) or on Luria-Bertani agar (LA). For antibiotic selection, erythromycin (Erm) was used at 5 µg/ml for GAS and 500 µg/ml for *E. coli*.

**Allelic exchange mutagenesis** was performed essentially as previously described [32]. The *ropB* allele plus upstream and downstream flanking regions was amplified from 5448 or 5628 using the primers RopB-F-BamHI (5′-CAG GAT CCC TCA TTT CAG TTG ACA AGA AAC-3′) and RopB-R-XbaI (5′-CGC TCT AGA TAC CAA AAG GCT AGA CCT CTG-3′). The PCR products and temperature sensitive vector pHY304 were ligated using T4 ligase to create the plasmids pHY5448RopB and pHY5628RopB. The plasmid pHY5628RopB was transformed into GAS strain 5448 and the plasmid pHY5448RopB was transformed into GAS strain 5628, and Erm^r^ transformants were grown at the permissive temperature for plasmid replication (30°C). Single-crossover chromosomal insertions were selected by shifting to the nonpermissive temperature (37°C) while maintaining Erm selection. Single crossover colonies were then grown in the absence of antibiotic selection at 30°C and Erm^s^ colonies were then screened for the presence of the appropriate *ropB* allele using DNA sequence analysis. Confirmed allelic exchange mutants were designated 5448R− (5448 containing the mutant *ropB* allele from 5628) and 5628R+ (5628 containing the wildtype (WT) *ropB* allele from 5448).

### SpeB activity assays

Cysteine protease activity assays were performed as described by Collin *et al.* [33]. GAS strains were grown overnight at 37°C. Cultures were then diluted 1∶50 and grown for 17 h at 37°C. The cultures were centrifuged at 3200×*g*, and the supernatants sterile-filtered through 0.2 µm syringe-driven filters (Whatman). 200 µl of filtered supernatant was mixed with 200 µl of activation buffer (1 mM EDTA, 20 mM DTT in 0.1 M sodium acetate buffer, pH 5.0) and incubated for 30 min at 40°C. 400 µl of 2% (w/v) azocasein in activation buffer was then added and incubated for a further 1 h at 40°C. Trichloro-acetic acid was then added to a final concentration of 15% (w/v) and thoroughly mixed. The mixture was then centrifuged at 15,000×*g* for 5 min and the OD_366_ of the supernatant was then measured to indicate cleavage of the azocasein by SpeB.

### SpeB Western blot

For Western blot analysis, bacterial cultures were grown to late stationary phase (17 h) and pelleted by centrifugation at 3,200×*g* for 10 min. The supernatants were sterile-filtered through 0.22 µm syringe-driven filters (Millipore). Supernatants were diluted 1∶8 and 10 µl of each sample was run on a 10% Bis-Tris Gel with MOPS running buffer (Invitrogen). Proteins were then transferred to a Nitrocellulose membrane (Invitrogen) by use of a Trans-Blot SD semi-dry transfer cell (BioRad) for 1 h at 20 V. The presence of SpeB in culture supernatants was detected using primary rabbit anti-SpeB diluted 1∶1,000 for 1.5 h. Following subsequent washing, the membrane was incubated with a secondary goat anti-rabbit-HRP conjugate diluted 1∶10,000 for 1 h. SuperSignal West Pico Chemiluminescent Substrate (Thermo Scientific) was used to expose autoradiography film as per the manufacturer's instructions.

### Hemolytic activity assay

Fresh, heparinized human blood from healthy volunteers was washed twice with sterile PBS and resuspended to a final concentration of 2% (v/v) in PBS. Bacterial cultures were grown to mid-log phase (OD_600_ = 0.4), pelleted by centrifugation at 3,200×*g* and the supernatants sterile-filtered through 0.22 µm syringe-driven filters (Millipore). 100 µl of blood solution was mixed with 100 µl of serially diluted supernatant. The plates were incubated for 1 h at 37°C, then 1 h at 4°C. Following centrifugation at 1,500×*g* for 10 min, 100 µl was transferred into a fresh 96-well flat bottom plate and the absorbance at 405 nm was recorded.

### DNase activity assays

DNase assays were performed as previously described [34], [35]. Briefly, GAS strains were grown to mid-log phase (OD_600_ = 0.4), and the supernatants collected by centrifugation at 3200×*g*. 2.5 µl of bacterial supernatant was added to 7.5 µl of calf thymus DNA (1 µg/ml) and 40 µl of DNase buffer (3 mM MgCl_2_, 3 mM CaCl_2_, 300 mM Tris; pH 7.4). The reaction was incubated for 5 min at 37°C and then stopped by the addition of 12.5 µl 0.33 M EDTA (pH 7.3). The relative DNA degradation was then compared by gel electrophoresis on a 1% agarose gel.

### Neutrophil killing assays

Human neutrophils were isolated from venous blood of healthy volunteers using the PolyMorphPrep kit as per the manufacturer's instructions (Axis-Shield, Norway). In 96-well plates, we mixed 2×10^5^ neutrophils and 2×10^4^ colony forming units (cfu) of logarithmic phase GAS in RPMI containing 2% heat-inactivated autologous plasma in a total volume of 200 µl. The plates were centrifuged at 500×*g* for 5 min and incubated at 37°C in 5% CO_2_ for 30 min. The neutrophils were then hypotonically lysed in H_2_O, serially diluted and plated on THA. The plates were incubated overnight at 37°C and cfu enumerated. Control wells containing bacteria but no neutrophils were used to determine survival. Percentage survival was calculated as [cfu/ml experimental well]/[cfu/ml control well]×100%.

### *In vivo* SpeB switching studies

GAS strain 5628R+ was grown to mid-log phase (OD_600_ = 0.4), pelleted by centrifugation at 3,200×*g* and washed twice with sterile PBS. Bacteria were then resuspended in PBS at a final concentration of 1×10^9^cfu/ml. 100 µl of bacterial suspension (1×10^8^ cfu) was injected subcutaneously into the flank of 10 week old C57BL6/J mice. Three days post-infection, bacteria were recovered from the lesion and screened for SpeB status using the skim-milk agar method as previously described [36].

### Tissue cage implantation and *in vivo* bacterial growth

To obtain *in vivo*-derived RNA for microarray analysis, a previously described subcutaneous murine Teflon chamber model was used [31]. Approximately 10^8^ cfu of bacteria were injected in sterile Teflon chambers that had been surgically inserted under the skin of age-matched female BALB/c mice. After 24 h, bacteria were collected, tested for purity and phenotypic homogeneity on blood agar plates and Columbia-casein agar plates, respectively. Only pure isolates with homogeneous protease activity (either positive or negative) were further selected for RNA extraction. Selected isolates recovered from Teflon chambers were centrifuged, and their pellets were washed twice in PBS, sheared with beads (QBiogene), then processed for RNA extraction according to the RNEasey protocol (Qiagen).

### Expression microarrays

For transcriptome analysis, we used oligomer-based microarrays printed in the Molecular Resource Center at the University of Tennessee Health Science Center. Each array consists of duplicates of 2,328 oligomers (70-mers) that represent all ORFs in M1 GAS, strain SF370 (GenBank accession # NC_002737) in addition to oligomers representing ORFs from prophages in strains MGAS8232 (GenBank accession# NC_003485) and MGAS315 (GenBank accession # NC_004070). The arrays also contained positive and negative controls of streptococcal ribosomal DNA and alien DNA (Stratagene), respectively.

### cDNA preparation and microarray hybridization

Bacterial RNA was treated with DNase Turbo (Ambion) for 1 h to remove any genomic DNA contamination, then converted it to dendrimer-labeled cDNA using the 3DNA Array 900TM kits (Genisphere, http://www.genisphere.com/array_detection_900.html) following the manufacturer's protocol. Equal amounts of dendrimer-labeled cDNA from different pairs of isolates were mixed, applied to the glass microarrays, and incubated for 16 h. After this first hybridization, the arrays were washed, labeled with a mixture of Alexa Fluor 546 and Alexa Fluor 647 (Genisphere), incubated for 3 h, washed again, then scanned bv GenePix 4000B scanner (Axon Instruments, Inc.) We followed a cyclic design that allowed us to compare every condition to each other at least twice, and guaranteed dye swapping to eliminate effects of non-specific binding.

### Analysis of microarray data

To analyze the microarray data, GenePixPro 4.0 software (Axon Instruments, Inc.) was used for image processing, fluorescent normalization and spot finding, then GeneSpring GX 7.3.1 (Agilent Technologies) was used for normalization, statistical analysis, clustering analysis and gene-list generation. All primary microarray data were submitted to the NCBI Gene Expression Omnibus (GEO) in accordance with MIAME standards (GEO accession # GSE13656).

### Murine systemic infection models

GAS strains were grown to mid-log phase (OD_600_ = 0.4), pelleted by centrifugation at 3,200×*g* and washed twice with sterile PBS. For intravenous challenge, bacteria were then resuspended in PBS at a final concentration of 1×10^9^cfu/ml. 200 µl of bacterial suspension (2×10^8^ cfu) was injected into the lateral tail vein of 10 week old C57BL6/J mice. For intraperitoneal infection, the bacteria were resuspended at a concentration of 2.5×10^8^ cfu/ml in PBS containing 5% (w/v) mucin. 200 µl of bacterial suspension (5×10^7^ cfu) was injected into the peritoneal cavity of C57BL6/J mice. For both experiments, the mice were monitored for 10 days and deaths recorded every 24 h.

## Results

### Sequence analysis of clinical isolate 5628 reveals intact *speB* and *covR/S* but mutation in *ropB*

**Figure 1:**
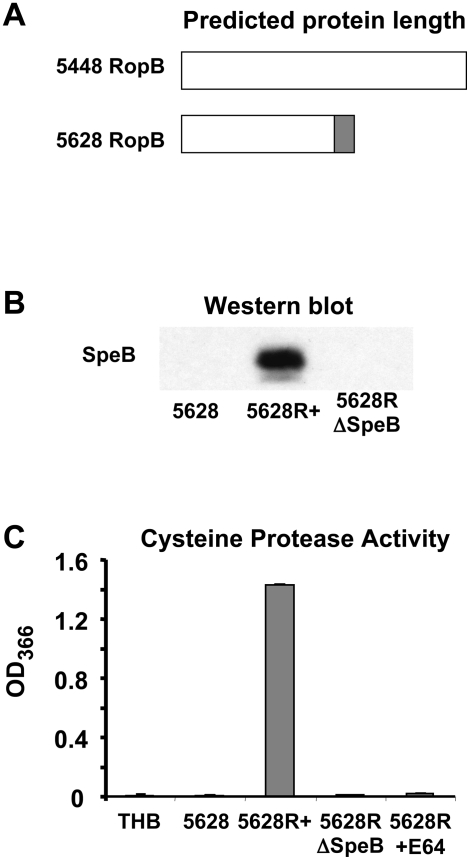
Mutation in *ropB* results in truncation of the RopB protein and abrogation of SpeB expression. (A) Schematic representation of the RopB protein expressed by GAS strains 5448 and 5628. In 5628, white region represent homology to 5448, shaded region indicates unique protein sequence in 5628. (B) Western blot for SpeB using overnight culture supernatants. (C) SpeB activity assay using azocasein substrate.

M1T1 GAS clinical isolate 5628 was found on screening to lack SpeB activity by azocasein assay. DNA **s**equencing was performed using primers listed in Table 1. No mutations were found in the strain 5628 in the *speB* gene nor in the *covR/S* locus, which has been previously linked to loss of SpeB expression [37], [38]. Further sequencing was performed on the previously described regulators *luxS*, *rofA*, *ropA* and *ropB* [24], [39]–[41]. This analysis revealed a point mutation in *ropB*, leading to truncation of RopB at amino acid 170 of the 280 amino acid protein (Fig. 1A). The truncation of RopB suggested that this may be the cause of lack of SpeB expression in this strain.

**Table 1:** Primers used for sequencing the *ropB* locus (including *speB*), the *covR/S* locus, *luxS*, *rofA* and *ropA*.

Primer name	Sequence (5′–3′)
RopB-F1	GACTGTTCGTTAGAAAGCCA
RopB-F2	CTCCTGATACGATGATAA
RopB-F3	AAAGTTTTCTTTCAAGGC
RopB-F4	CTTTGATTTGTTCGACAT
RopB-F5	TACCATGAATGGTAATAG
RopB-F6	TATCTCACTACCATTTTGC
RopB-F7	TGAGTTTCTCTTTATTAG
RopB-F8	GAACGGTGTTGTGTGTCT
RopB-R9	AGTCACCCATTGATAAAG
RopB-R10	AGGCGGCTTCAACGGTTA
RopB-R11	AGACTACACTTACACACT
RopB-R12	ATCCAAAAATCAGCAGCTATC
RopB-R13	TTAACAAAATGAGAACGG
RopB-R14	TGATAGTCGCTTATGATA
RopB-R15	CATATTGACAAACATCCGAATCG
RopB-R16	GCTGTTGAGATAAACTAC
RopB-R17	CTAGACCTCTGCTCACTAG
CovRS-F1	GCTATTCCGGTACAGGTGT
CovRS-F2	GTCAATGGTCGTGAAGGGT
CovRS-F3	GATGTCTATATTCGTTATCTCC
CovRS-F4	GATGATTTTTACCACAGATAAC
CovRS-F5	GCATATTGGTCTCTTACAAC
CovRS-F6	GCAAATTGTAGATGGGTATCA
CovRS-R7	GCGGAAAATAGCACGAATAC
CovRS-R8	AGGCAATCAGTGTAAAGGCA
CovRS-R9	CTTGTGCCAAATAACTCAACA
CovRS-R10	ATCAAAAGCCTGCTCAAATGA
CovRS-R11	CTTTCATGTCATCCATCATTG
CovRS-R12	TTGCTCTCGTGTGCCATCT
LuxS-F1	GCAGCTCTATTGCACCTAT
LuxS-F2	AAGAAGTTATCGTCGAAA
LuxS-F3	AATCCTACTGACCTATTT
LuxS-R4	TAGTGGCAACACGGTGAA
LuxS-R5	TGAAAACCTGTTCGACAG
LuxS-R6	ATAATGGCAATGGTTAC
LuxS-R7	GTACCTTACAATCAAGATGTT
RofA-F1	TCTTGAGCTAATGCAACCGT
RofA-F2	GAATCCGTTAGGAGATGA
RofA-F3	GTTTCGATAATATCATGG
RofA-F4	AAAGGATGTGTAAATTGG
RofA-F5	ACAAGGTTTCCAAATAAG
RofA-R6	AAGCAATTAACATAAGCG
RofA-R7	TCTGCAACATTTTATTCC
RofA-R8	GGCATTAAAGTTTATGAC
RofA-R9	TAGGAAGAGAGGTCCCTT
RofA-R10	GAACTTGAATCTGGATTTATTG
RopA-F1	TCTTGTCCTGCAAATACGTC
RopA-F2	TCTTCTTGAGTTGTACCA
RopA-F3	CTCAACACCATCAACTGA
RopA-F4	GATTTGTAGCTTTGTTTTC
RopA-R5	TTAAGGAACAAAACGTACAAG
RopA-R6	ATGTTGACACACTTGAAG
RopA-R7	TGTTGTGTCAATGGAAAA
RopA-R8	ATGCCATAGTCATCCGTT
RopA-R9	AATCCTTTCTTTGATAGTTTATC

### Repair of the 5628 *ropB* allele restores SpeB expression and activity

Western blot detected SpeB in overnight culture supernatants of 5628R+, containing the intact *ropB* allele, but not in the clinical isolate 5628 nor the isogenic mutant 5628RΔSpeB, containing the intact *ropB* allele but lacking *speB* (Fig. 1B). This expression correlated with a restoration of extracellular protease activity as detected by azocasein degradation assay (Fig. 1C). Protease activity was abrogated by the addition of the cysteine protease inhibitor E64. These data demonstrated that the *ropB* point mutation was indeed responsible for the lack of SpeB expression in the clinical isolate 5628.

### *RopB* mutation does not affect bacterial growth, hemolysis, DNase activity or resistance to neutrophil killing

**Figure 2:**
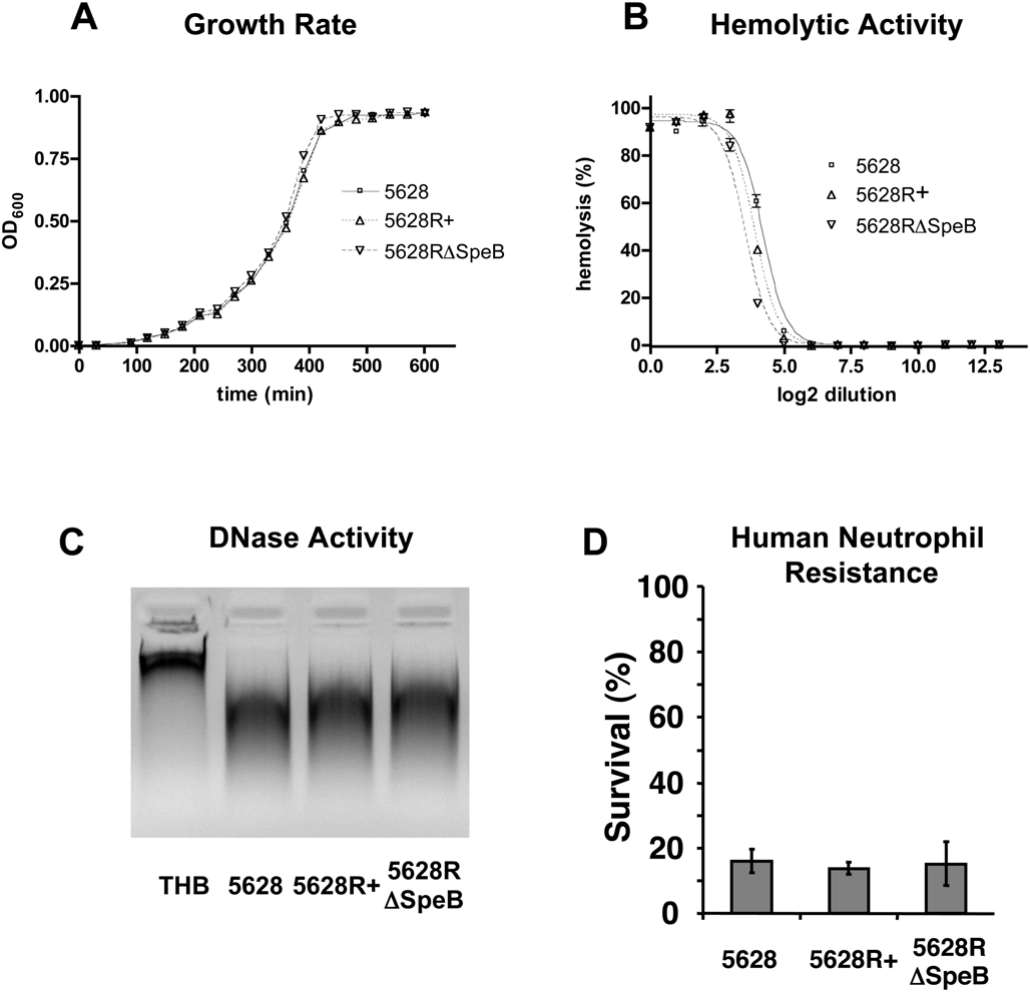
RopB mutation does not affect *in vitro* growth, hemolytic activity, extracellular DNase activity or resistance to neutrophil killing. (A) OD_600_ of *in vitro* grown bacterial cultures in THB. (B) Hemolysis of red blood cells by bacterial culture supernatants. (C) Degradation of calf thymus DNA by bacterial culture supernatants, run on a 1% agarose gel. (D) Bacterial survival after 30 min incubation with human neutrophils.

GAS strain 5628 and its isogenic mutants 5628R+ and 5628RΔSpeB were grown in THB, and the OD_600_ was measured over time. No significant difference was found among the growth rates of the three bacterial strains (Fig. 2A). Hemolysis assays were performed to determine the effect of *ropB* mutation on the cytolytic ability of GAS. No significant difference was seen in hemolytic activity among strains containing the WT or mutated *ropB* allele (Fig. 2B). Extracellular DNase activity of mid-log phase bacteria and GAS resistance to neutrophil killing were also unaffected by mutation in *ropB* (Fig. 2C and 2D).

### SpeB-positive bacteria revert to a SpeB-negative phenotype on subcutaneous infection

C57BL6/J mice were subcutaneously infected with the SpeB-positive strain 5628R+. Three days post-infection, bacteria were recovered from the lesion and screened for SpeB status using the skim-milk agar method. While the inoculum was 100% SpeB-positive, bacterial populations recovered from the lesions of individual mice were found to be 2%, 4%, 6%, 8%, and 24% SpeB-negative. Five representative colonies were picked, and the *covR/S* locus was sequenced in each of them. All colonies sequenced were found to have substitution mutations in *covR/S* resulting in truncation of CovS. This finding reveals that when SpeB-negative colonies are selected for *in vivo*, this selection is predominantly a phenomenon associated with CovR/S inactivation. The *ropB* mutation of strain 5628 was not recapitulated on passage in the mouse subcutaneous infection model.

### SpeB is the principal target of RopB regulation *in vivo*

**Figure 3:**
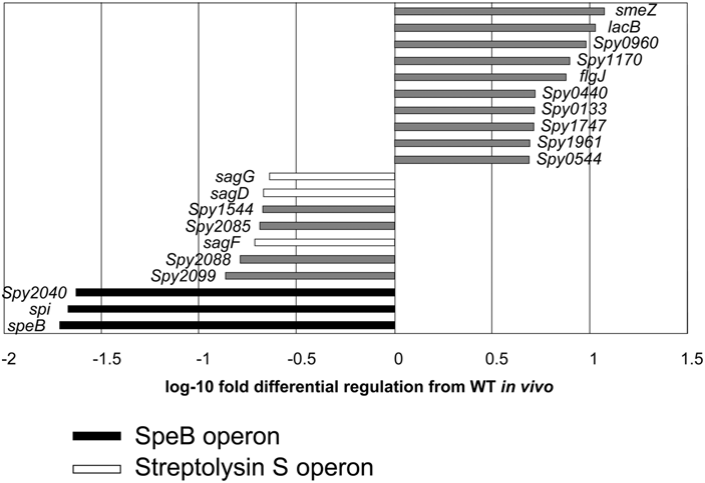
Microarray analysis emphasizes the down-regulation of the SpeB operon. The figure shows the top 10 genes up- and down-regulated in the *ropB* mutant bacteria compared to WT when the bacteria are inoculated *in vivo* (P<0.05). The values plotted represent log ratios (log mutant/WT), and genes co-clustered in operons are highlighted (black bars, SpeB operon; white bars, SLS operon).

Microarray analysis was performed on *in vivo*-derived RNA from the well characterized GAS strain 5448 and its derivative 5448R−, containing the mutated *ropB* allele from 5628. Fourty-seven genes were found to be down-regulated and 52 genes were found to be up-regulated in 5448R− greater than 2-fold (P<0.05). The most strongly down-regulated genes in 5448R− are members of the SpeB operon, as would be expected from the lack of SpeB expression and activity in 5628 (Fig. 3). In addition to the SpeB operon, genes of the streptolysin S operon were also found to be strongly down-regulated in the *ropB* mutant strain. Apart from the gene encoding the superantigen SmeZ, there was an absence of virulence-related genes found to be strongly up-regulated in the strain 5448R−, which expressed the truncated RopB.

### RopB is required for virulence in systemic infection

**Figure 4:**
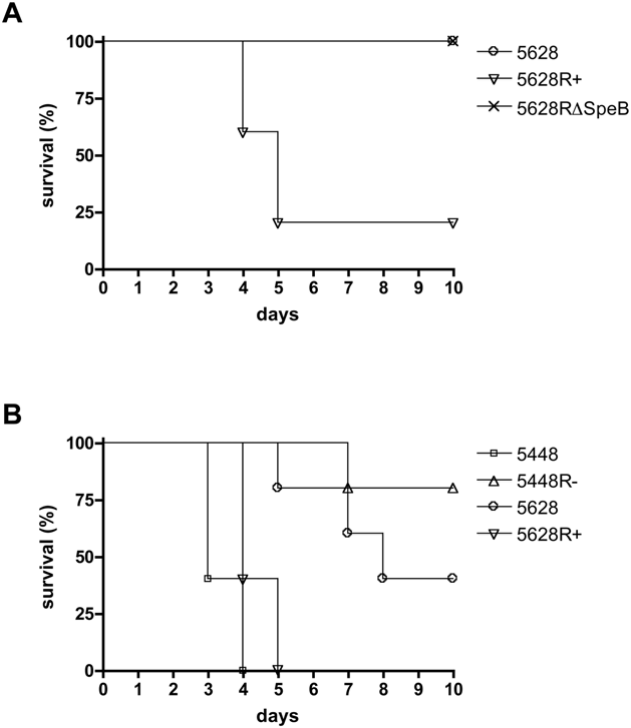
RopB and SpeB-negative bacteria show reduced virulence in systemic infection models. (A) Intraperitoneal infection of C57BL6/J mice with 5×10^7^ cfu of GAS strains with 5% mucin. (B) Intravenous challenge of C57BL6/J mice with 2×10^8^ cfu of GAS strains.

To determine the effect of *ropB* mutation on virulence, C57BL6/J mice were subjected to intraperitoneal infection with mid-logarithmic phase bacteria. GAS strain 5628R+ expressing a full length RopB showed increased virulence compared to the strain 5628 with a truncated RopB (Figure 4A). Strain 5628RΔSpeB containing a full-length RopB but lacking SpeB also showed reduced virulence, suggesting that the reduced virulence of 5628 may primarily result from the lack of SpeB expression in this strain. Virulence was also examined in an intravenous model of systemic infection. The well characterized strain 5448 and its isogenic mutant 5448R−, containing the *ropB* allele from 5628, were further included to examine the effect of this allelic variation in a well-characterized virulent strain of GAS. The two strains containing a truncated RopB (5628 and 5448R−) demonstrated similarly reduced virulence compared to the two strains containing a full-length RopB (5628R+ and 5448) (Fig. 4B), confirming that inactivation of RopB results in decreased virulence in systemic infection.

## Discussion

The cumulative contribution of the secreted cysteine protease SpeB to the pathogenesis of invasive GAS infection is at present unclear, and studies in various GAS serotypes and animal models have produced varying results [19]–[21]. In the globally-disseminated clonal M1T1 serotype associated with epidemic invasive GAS infection, an inverse relationship has been found between SpeB activity and clinical disease severity [8]. Recently, we and others have found a connection between inactivation of SpeB through mutation in the two-component regulatory system covR/S and development of invasive disease in the murine model [37], [38]. In this study, we have investigated a second mechanism of inactivation of SpeB, namely through truncation of the regulator RopB. We have shown that in M1T1 GAS, when the *covR/S* locus is intact and DNase Sda1 activity is unaffected, *ropB* point mutation results in reduced virulence *in vivo*, despite inactivation of SpeB activity.

The SpeB-negative, serotype M1T1 clinical isolate 5628 was used to investigate the role of RopB in virulence. RopB has previously been shown to be necessary for SpeB production [23], [24] as well as regulation of other GAS virulence factors [25]–[29]. The M1T1 strain 5628 contains a point mutation in the *ropB* gene that results in a truncation of the last 110 amino acids of the 280 amino acid protein. Restoration of full-length RopB in 5628R+ resulted in gaining SpeB expression, but did not result in changes in hemolysis, DNase activity or resistance to killing by neutrophils. *RopB* mutation in this particular strain does not result in the kind of global phenotypic change found with *covR/S* mutation where differential expression was observed of multiple genes encoding virulence determinants, including *sic*, *ska*, *slo*, *speA*, *speJ*, *scpC* and the hyaluronic acid synthesis operon [37] which were all unaffected in the *ropB* mutant examined in this work.

Recently, we have demonstrated that WT, SpeB-positive bacteria undergo a phase-shift to a SpeB-negative phenotype after subcutaneous infection of mice [17]. This phase switch was observed to be the result of mutations in the two-component regulatory system *covR/S* [38]. CovR/S is a global regulator, whose inactivation is linked to an invasive phenotype and involves up-regulation of many genes encoding virulence factors such as streptodornase, streptokinase, streptolysin O, streptococcal inhibitor of complement and the hyaluronic acid capsule synthesis operon [37]. Of the down-regulated genes in CovR/S mutant strains, SpeB would appear to be one of the most important due to its ability to degrade many host and self-proteins. It is the loss of SpeB expression in these mutant bacteria that is hypothesized to allow the invasive spread of GAS by sparing from SpeB degradation self-proteins involved in plasminogen accumulation and activation [42]. In this study we found that SpeB-positive bacteria with a restored RopB reverted to a SpeB-negative phenotype on subcutaneous infection. Furthermore, the switch to a SpeB-negative phenotype appeared to occur exclusively through mutations in *covR/S*. This result suggests that *covR/S* mutations are the predominant method of phenotypic switching in GAS and that *ropB* mutation is not readily selected for *in vivo*. This leads to the conclusion that *ropB* mutation in 5628 does not represent a similar mechanism to *covR/S* mutation and may (a) be an incidental occurrence or (b) arise secondary to different selection pressures than those associated with the shift to invasive infection.

Microarray analysis was conducted using *in vivo*-derived RNA, since the transcriptome of bacteria grown *in vitro* may differ greatly from the transcriptome found during the course of infection. Implantation of tissue cages allows for bacteria to be grown in an *in vivo* environment for 24 hours prior to recovery and RNA extraction. The microarray data showed members of the SpeB operon to be the most strongly down-regulated genes following inactivation of RopB. This finding supported the *in vitro* and *in vivo* data that suggested SpeB is the main target of RopB regulation in this strain.

Virulence studies utilizing systemic models of infection showed that a full-length RopB was required for virulence. The clinical isolate 5628 expressing a truncated RopB, as well as the allelic exchange mutant 5448R− also expressing the truncated RopB from 5628, showed reduced virulence compared to strains containing a full-length RopB. These data illustrate the necessity for an intact RopB for full virulence *in vivo*. In addition, the strain 5628RΔSpeB expressing an intact RopB but lacking SpeB showed similarly reduced virulence to the strain with a truncated RopB. While the down-regulation of the streptolysin S operon may contribute to the reduced virulence, this finding implies that the loss of SpeB in this strain is the main cause of the lack of virulence exhibited. This finding leads to the conclusion that inactivation of SpeB alone is not sufficient to initiate invasive disease. Moreover, these data suggest that SpeB loss, only in the context of mutation of the *covR/S* regulatory circuit, promotes invasion. Of note, the additional virulence factors streptokinase and M1 protein, are both up-regulated in *covR/S* mutant M1T1 strains [37], but not in the *ropB* mutant under investigation in this study. Streptokinase and M1 protein are believed to play a role in the accumulation of host plasmin at the GAS cell surface, a process which is thought to accentuate invasive disease [5], [42], [43].

Together these data provide evidence of RopB's role in virulence. In M1T1 GAS, an intact RopB and efficient SpeB production are necessary for systemic infection.
